# Characterization and anti-aging activities of polysaccharide from *Rana dybowskii* Guenther

**DOI:** 10.3389/fphar.2024.1370631

**Published:** 2024-03-28

**Authors:** Yiping Li, Xuyan Zhao, Jing Wang, Qi Yu, Jing Ren, Ziye Jiang, Lili Jiao

**Affiliations:** ^1^ College of Pharmacy, Changchun University of Chinese Medicine, Changchun, China; ^2^ The Affiliated Hospital Changchun University of Chinese Medicine, Changchun University of Chinese Medicine, Changchun, China; ^3^ Jilin Ginseng Academy, Changchun University of Chinese Medicine, Changchun, China

**Keywords:** *Rana dybowskii* Guenther, polysaccharide, structural characterization, anti-oxidant activity, anti-aging

## Abstract

**Introduction:** *Rana dybowskii* Guenther (RDG), as a traditional Chinese medicine, has been shown to have antioxidant effects. However, studies on the anti-aging effect of RDG are still limited.

**Methods:** In this study, we prepared polysaccharides from the skin of RDG (RDGP) by hot water extraction, alcohol precipitation, ion-exchange chromatography and gel chromatography. The proteins were removed using the Sevage method in combination with an enzymatic method. The structural features were analyzed using high-performance gel permeation chromatography, β-elimination reaction and Fourier transform infrared spectra. The anti-aging effect of RDGP was investigated by using D-Gal to establish an aging model in mice, and pathological changes in the hippocampus were observed under a microscope.

**Results:** We obtained the crude polysaccharide DGP from the skin of RDG, with a yield of 61.8%. The free protein was then removed by the Sevage method to obtain DGPI and deproteinated by enzymatic hydrolysis combined with the Sevage method to further remove the bound protein to obtain the high-purity polysaccharide DGPII. Then, DGPIa (1.03 × 10^5^ Da) and DGPIIa (8.42 × 10^4^ Da) were obtained by gel chromatography, monosaccharide composition analysis showed that they were composed of Man, GlcA, GalNAc, Glc, Gal, Fuc with molar ratios of 1: 4.22 : 1.55: 0.18 : 8.05: 0.83 and 0.74 : 1.78: 1: 0.28: 5.37 : 0.36, respectively. The results of the β-elimination reaction indicated the presence of O-glycopeptide bonds in DGPIa. The Morris water maze test indicated that mice treated with DGPIIa exhibited a significantly shorter escape latency and increased time spent in the target quadrant as well as an increase in the number of times they traversed the platform. Pathologic damage to the hippocampus was alleviated in brain tissue stained with hematoxylin-eosin. In addition, DGPIIa enhanced the activities of SOD, CAT, and GSH-Px and inhibited the level of MDA in the serum and brain tissues of aging mice.

**Discussion:** These results suggest that RDGP has potential as a natural antioxidant and provide useful scientific information for anti-aging research.

## 1 Introduction

Aging is a multifactorial and all-round degenerative process including cognitive decline, which is an important factor in the occurrence of most human diseases (Zhu et al., 2020). Numerous studies have linked aging to a decline in antioxidant status. The free radical doctrine suggests that the main factor leading to aging of the body is oxidative damage caused by reactive oxygen species (ROS). Although normal cells require the production and removal of reactive oxygen species, this balance can be disrupted under pathogenic conditions, when there is an excess of reactive oxygen species, proteins, lipids, nucleic acids and other biological macromolecules will suffer damage (Zhang et al., 2021). To avoid cell damage, organisms have a complex antioxidant system available for scavenging lipid peroxides and free radicals, which consists of catalase (CAT), superoxide dismutase (SOD), glutathione peroxidase (GSH-Px), and some trace elements and vitamins (Yang et al., 2019). Some synthetic compounds have strong free radical scavenging effects, but they usually have side effects (Jiao et al., 2014), so work is ongoing to develop a natural, nontoxic and effective antioxidant.

Polysaccharides, as an important part of living organisms, are natural macromolecules found in animals and plants. Numerous studies have shown that polysaccharides have antioxidant and anti-aging activities and are noted for their low toxicity as natural antioxidants (Hou et al., 2020). Polysaccharides exert antioxidant effects through mechanisms such as increasing the activities of antioxidant enzymes and reducing oxidative stress. There has been a great deal of research regarding the antioxidant effects of plant polysaccharides and fungal polysaccharides (Fernandes and Coimbra, 2023), but a growing body of research suggests that animal polysaccharides may also be potential candidates for antioxidants. Xiang et al. extracted a water-soluble polysaccharide with antioxidant capacity from mussel, a mollusk, that can protect cells from oxidative stress (Xiang et al., 2022). Polysaccharides extracted from starfish by Zhang et al. presented excellent antioxidant effects (Zhang et al., 2013). Ramasamy et al. prepared chitosan from chitin extracted from the cuttlebone of Sepia kobiensis and demonstrated that it can act as a natural antioxidant (Ramasamy et al., 2014).

RDG is a comparatively small anuran, mainly from the northeastern region of China, containing a variety of nutritious substances such as sugars, amino acids, fats and peptides. Toad oil, extracted from the dried oviducts of female frogs, is a very valuable health product, RDG oil is also known as “soft gold” (Wang et al., 2015a). Polysaccharides are one of the important components of RDG, which has multiple biological activities and high utilization and development value. Previous researchers extracted a simple polysaccharide free of sulfates, proteins, and aldehydes from Chinese forest frogs, consisting of glucose, galactose, and mannose, with a molecular weight of 12.8 kDa, and the results of their antioxidant activity assays indicated that the forest frog polysaccharide could be used as a novel natural antioxidant in food and pharmaceutical applications (Wang et al., 2015b). The main classes of polysaccharides in animals include glycosaminoglycans, chitin (Liu et al., 2023), glycogen (Adeva-Andany et al., 2016), etc. Glycosaminoglycans are negatively charged polysaccharides that are involved in a variety of biological processes (Sahu et al., 2023). In our study, we obtained a glycosaminoglycan from RDG, but its *in vivo* antioxidant effects as well as its anti-aging effects are unclear. Therefore, in this work, we extracted and purified high-purity polysaccharides from the skin of RDG, identified their structural properties by FT-IR, and investigated their anti-aging effects to provide theoretical references for the further development and utilization of RDG.

## 2 Materials and methods

### 2.1 Materials and reagents

Male ICR mice were provided by Changchun Yis Experimental Animal Technology Co., Ltd. and dried skin of *Rana dybowskii* Guenther, purchased from Taoyuan Forestry Farm, Shangying Forest Management Bureau, Shulan City, Jilin Province (127° 25′36.18″E; 44° 09′41.7″N; 338 m above sea level). Monosaccharide standards, including glucose (Glc), glucuronic acid (GlcA), N-acetylglucosamine (GlcNAc), galactose (Gal), galacturonic acid (GalA), N-acetylgalactosamine (GalNAc), mannose (Man), rhamnose (Rha), xylose (Xyl), arabinose (Ara), and fucose (Fuc), were purchased from Shanghai Yuanye Biotechnology Co. D-Galactose (D-Gal) was purchased from Sinopharm Chemical Reagent Co. Sepharose CL-6B (GE Healthcare), and DEAE-cellulose was obtained from Shanghai Yuanye Biotechnology Co. Assay kits for superoxide dismutase (SOD), malondialdehyde (MDA), catalase (CAT), and glutathione peroxidase (GSH-Px) were provided by Jiancheng Institute of Biotechnology, Nanjing, China. All other chemicals and reagents were of analytical grade.

### 2.2 General methods

Polysaccharide content was determined by the phenol-sulfuric acid method using glucose as a standard (Yang Y. et al., 2023). Protein and uronic acid were determined by the Bradford method and the m-hydroxybiphenyl method, respectively, bovine serum albumin and D-galacturonic acid were used as standards, respectively (Qi et al., 2022; Zhang et al., 2023). The sulfate content was determined by the barium chloride turbidimetric method using potassium sulfate as the standard (Liu et al., 2016). Homogeneity and molecular weight (*Mw*) were determined by using high-performance gel-permeation chromatography (HPGPC) (Zhang et al., 2009). Determination of monosaccharide composition using 1-phenyl-3-methyl-5-pyrazolone (PMP) derivatization and high-performance liquid chromatography (HPLC) (Liu et al., 2017). Fourier transform infrared (FT-IR) spectra were recorded in the range of 4000 and 500 cm^−1^ (Shakhmatov et al., 2014).

### 2.3 Extraction and purification of polysaccharides

**FIGURE 1:**
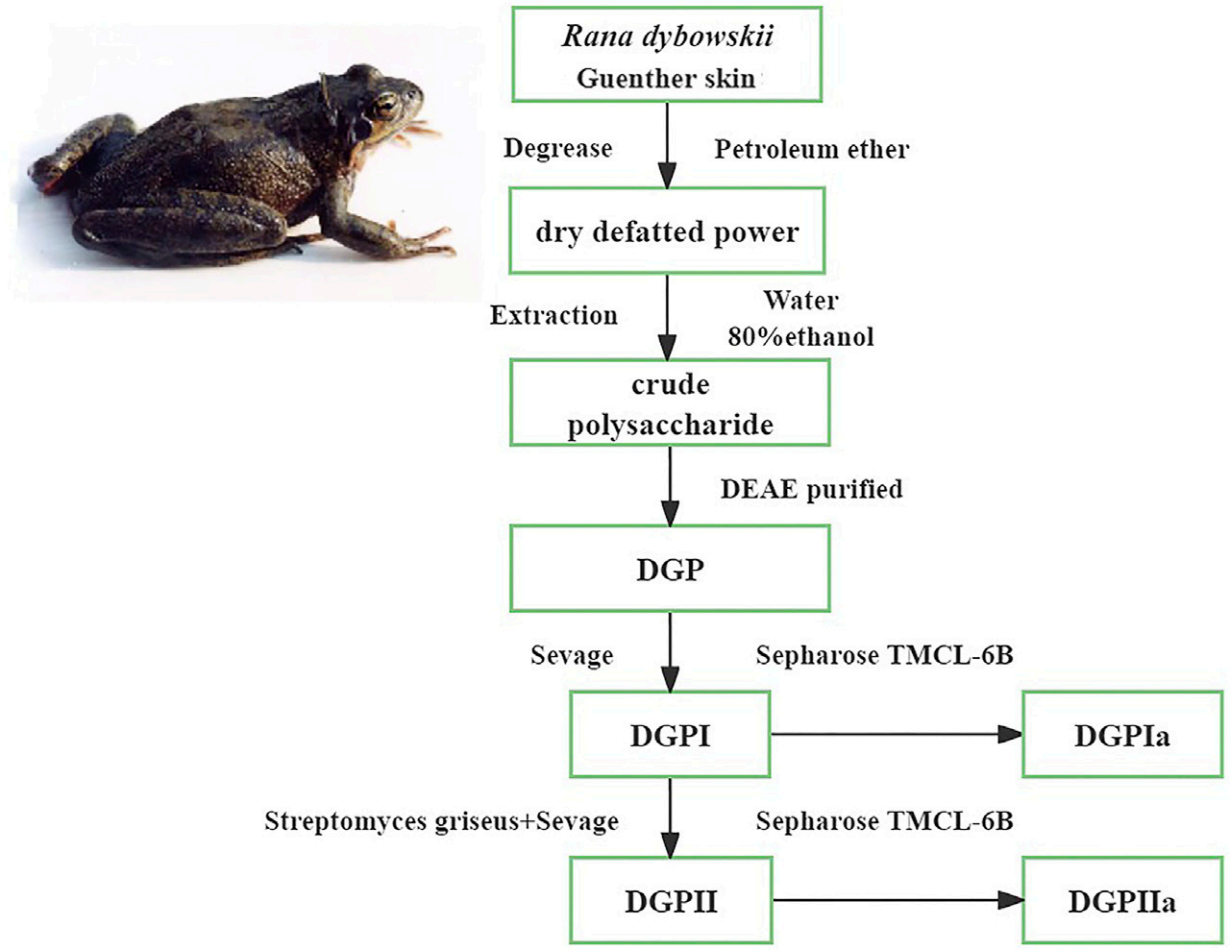
Procedure of the extraction and purification of polysaccharides from the *Rana dybowskii* Guenther.

RDG skin was degreased by soaking in petroleum ether at room temperature for 24 h, filtered and then lyophilized. After hot water extraction three times, 80% ethanol was added to the extraction solution and allowed to stand for 24 h at 4°C. The precipitate was subsequently collected by centrifugation (3500 rpm, 10 min) and freeze-dried to obtain crude polysaccharide. The crude polysaccharide was subjected to a DEAE-cellulose ion exchange column eluted with 1 M NaCl solution at a flow rate of 0.5 mL/min for 3–4 h to obtain DGP, which was deproteinated by Sevage reagent (chloroform:n-butanol = 4:1) to obtain the deproteinized polysaccharide (DGPI). In addition, DGPI was further purified by a combination of pronase and Sevage reagent to obtain the purified polysaccharide DGPII. DGPI and DGPII were dissolved in 10 mL of distilled water and applied to a Sepharose CL-6B gel column (2.5 ✕ 100 cm) and eluted with 0.9% NaCl solution at a flow rate of 0.5 mL/min. The sugar distribution was detected by the phenol‒sulfuric acid method, and the corresponding eluate was collected. Finally, the purified polysaccharides DGPIa and DGPIIa were obtained by dialysis, concentration and lyophilization. The process of separation and purification of each fraction is shown in Figure 1.

### 2.4 Judgment of glycopeptide bond type

Four milligrams of DGPIa was dissolved in 8 mL NaOH solution (0.2 mol/L). The mixture was reacted in a water bath for 2 h at 45°C, and then the reacted solution was detected using a UV spectrophotometer (TU-1810). The UV absorption values ranging from 190 nm to 400 nm were recorded. The UV absorption values of the sample solutions of the same concentration of DGPIa without alkali treatment were also measured in the same range.

### 2.5 Anti-aging activity *in vivo*

#### 2.5.1 Animal experiments

ICR male mice (8 weeks old, 23.0 ± 2.0 g) were housed at a temperature of 22°C–25°C and relative humidity of 40%–60% with a 12 h light/12 h dark cycle and a free-drinking diet. All experimental operations were performed in strict accordance with international guidelines for animal experiments and approved by the Ethics Committee of Changchun University of Chinese Medicine. After 3 days of adaptive feeding and weighing, 300 mg/kg of D-Gal was injected intraperitoneally. Fifty mice after D-Gal induction were randomly divided into six groups of 10 mice each: (1) Normal control group (0.9% physiological saline); (2) Model control group (0.9% physiological saline + D-Gal injection); (3) Positive control group (vitamin C, Vc) 300 mg/kg + D-Gal injection); (4) Low-dose DGPⅡa group (100 mg/kg + D-Gal injection); (5) Medium-dose DGPⅡa group (200 mg/kg + D-Gal injection); (6) High-dose DGPⅡa group (400 mg/kg + D-Gal injection). All groups of mice were housed under the same conditions and underwent continuous mold making for 7 weeks.

After the last treatment, the mice were fasted for 12 h, and blood was taken from the eye sockets. Blood samples were allowed to stand at 4°C for 30 min and then centrifuged at 3000 r/min for 15 min. The serum was collected, and the brain, thymus and spleen tissues were removed and washed with saline and stored at −80°C for analysis. The body weights were measured weekly during the whole experimental period. The thymus and spleen of mice were weighed, and the immune organ index was calculated as previously described (Zalys et al., 2000; Mao et al., 2009).

#### 2.5.2 Morris water maze

The Morris water maze is a classic behavioral test used to study learning memory in rodents, consisting of a circular pool, a platform that can be moved into position and hidden under the surface of the water, and an automatic image acquisition system. The water maze was placed in a uniformly lit, quiet environment. The pool of the water maze is 0.5 m high, the water temperature is controlled at approximately 25°C, and its interior is divided into four quadrants (Gacar et al., 2011).

Localization cruising-evasion latency is an important indicator of the Morris water maze, and its duration also represents the spatial learning memory ability of the animal. Each mouse was trained once a day for 120 s for 4 days prior to the official test. The mice were placed in a circular pool and allowed to find the jumping platform within 2 minutes, and if no platform was found, the mice were led to the jumping platform and waited for 10 s. The time it took for an animal to find a platform was called the escape latency. The formal experiment was started on Day 5 by placing the mice facing the pool wall and placing them from the opposite quadrant of the platform and recording the time they reached the platform. After the positioning navigation experiment, we started the space exploration experiment for mice, removed the underwater platform while marking the location and placed the mice into the water from the second quadrant. The mice were then observed for the time spent in the quadrant and the number of platform crossings within 120 s (Gómez-Padilla et al., 2020; Lissner et al., 2021).

#### 2.5.3 Antioxidant enzyme levels in serum and brain tissue

Mouse blood was collected and centrifuged at 4°C (3000 r/min) for 15 min, and serum was separated. A portion of the brain tissue was homogenized and centrifuged in a low-temperature environment, and then the supernatant was collected. The activities of SOD, CAT, GSH-Px, and MDA in serum and brain tissues were determined by using commercial kits according to the instructions (Jiancheng Institute of Biotechnology, Nanjing, China).

#### 2.5.4 Histopathological analysis

Under low temperature conditions, the mouse brain tissue was quickly removed, and the surface blood was washed with physiological saline, fixed in 4% paraformaldehyde and embedded in paraffin. The tissues were stained with hematoxylin-eosin (H & E), sealed with neutral gum after dewaxing, dewaxed and dehydrated, and placed in a 37°C thermostat overnight. The fabricated slides were evaluated for pathological changes under a 400x light microscope.

### 2.6 Statistical analysis

All data in this paper and in the figures are expressed as X ± S. Statistical software SPSS 23.0 was applied for ANOVA, and a *p* < 0.05 value was considered statistically significant.

## 3 Results and discussion

### 3.1 Purification and characterization

**FIGURE 2:**
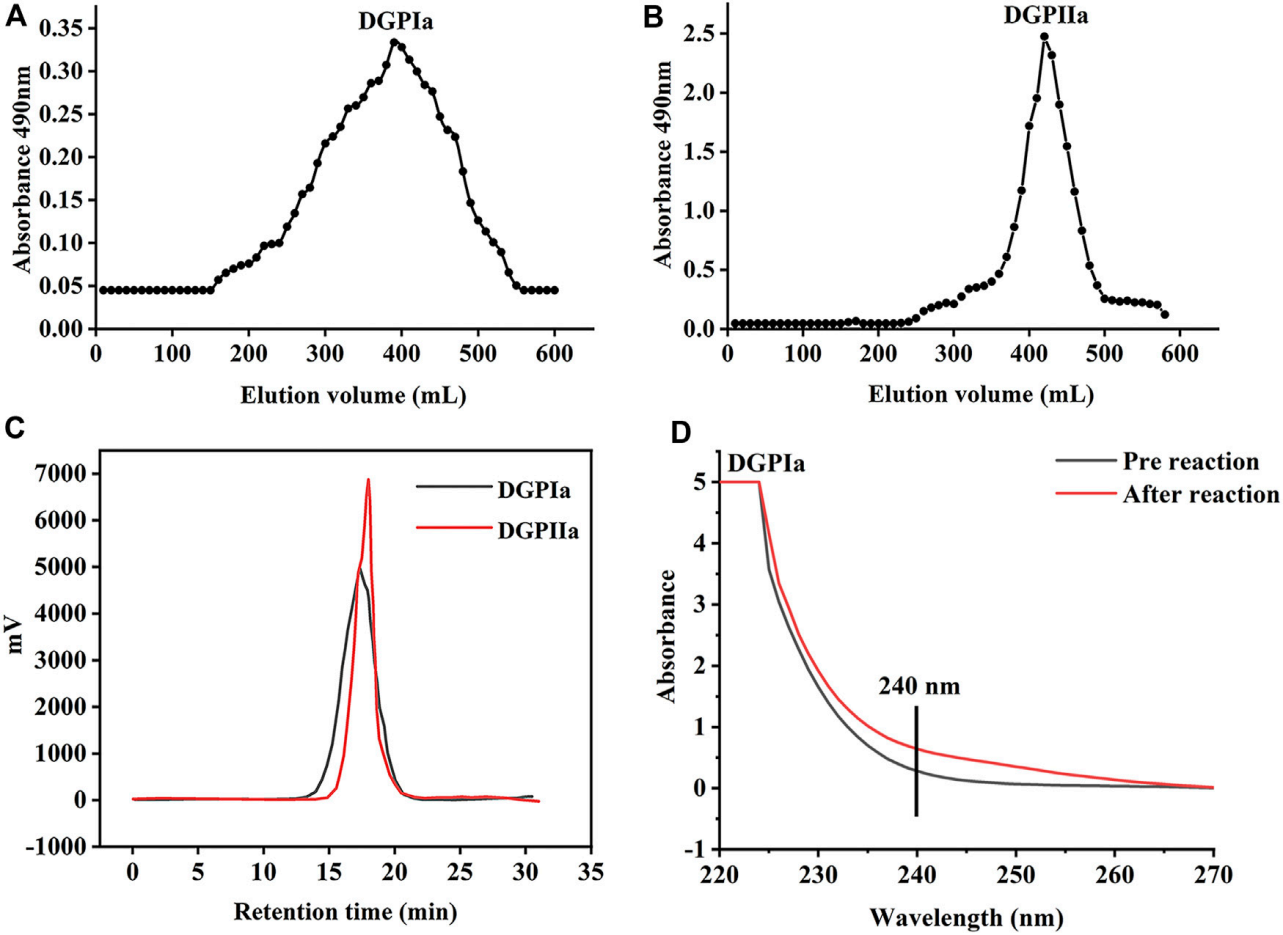
**(A)** The elution profiles of DGPIa on Sepharose TMCL-6B column; **(B)** The elution profiles of DGPIIa on Sepharose TMCL-6B column; **(C)** Molecular weight analysis of DGPIa and DGPIIa; **(D)** UV spectra of DGPIa with or without β-elimination by alkali treatment.

**FIGURE 3:**
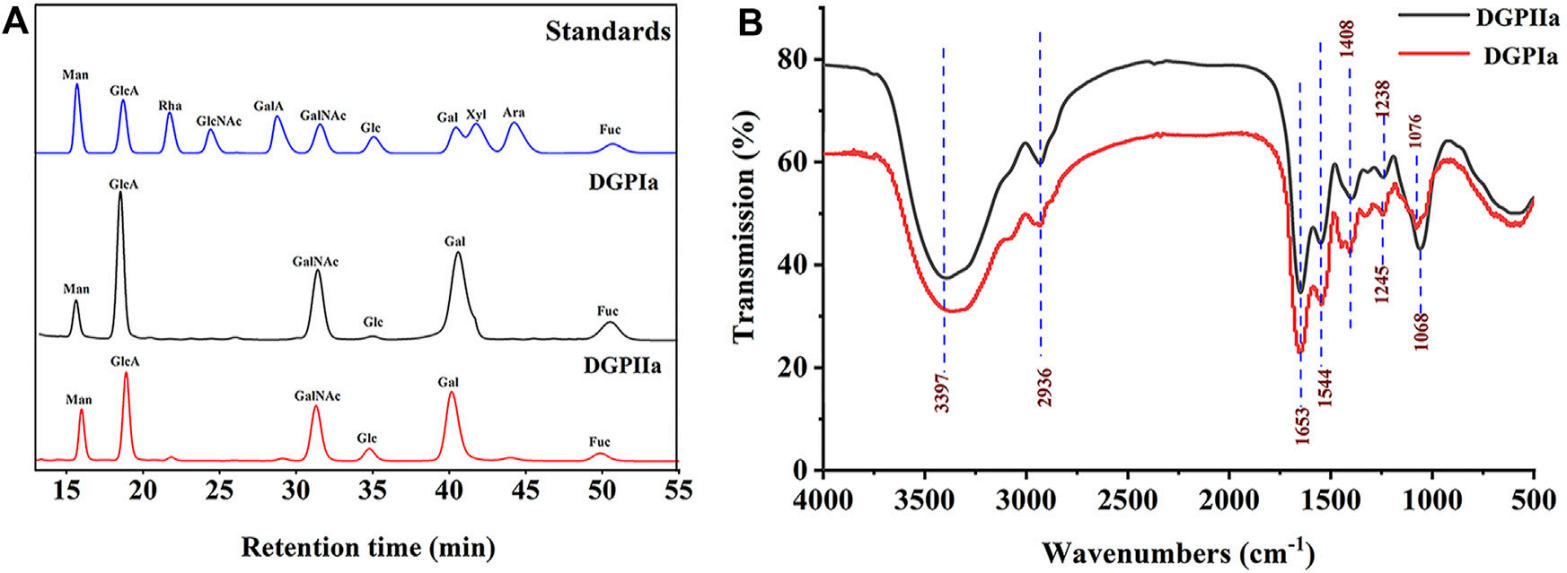
**(A)** Monosaccharide composition analysis of DGPIa and DGPIIa by HPLC; **(B)** FT-IR spectrum.

RDG skin polysaccharide DGP (514 mg) was obtained by hot water extraction and ion exchange. DGP was then purified by the Sevage method to obtain DGPI (360 mg). According to the results of the protein assay, DGPI still contained an amount of protein. Therefore, DGPI was further deproteinated by a combination of enzymatic methods and the Sevage method, and the purified polysaccharide DGPII (302 mg) was obtained. Afterward, DGPI and DGPII were fractionated by using a Sepharose CL-6B gel column (Figures 2A, B), and DGPIa (96 mg) and DGPIIa (83 mg) were obtained. The total sugar contents of DGPIa and DGPⅡa were 41.51% and 73.84%, respectively, as measured by the phenol sulfuric acid method. According to the Bradford method, the protein contents of DGPIa and DGPIIa were 5.18% and 0.64%, respectively, indicating that the protein in the polysaccharide had been completely removed. The uronic acid contents of DGPIa and DGPIIa were 22.62% and 25.93%, respectively. The sulfate contents of DGPIa and DGPIIa were determined to be 8.46% and 9.37%, respectively. And the chemical compositions of DGP, DGPI and DGPII are shown in Table 1. DGPIa and DGPIIa had a single symmetric peak in the HPGPC system (Figure 2C), indicating that they were homogeneous polysaccharides. According to the standard regression Equation Y = −0.3541X + 9.3927 (*R* ^2^ = 0.9967), the molecular weights of DGPIa and DGPIIa were calculated to be 8.42 × 10^4^ Da and 1.03 × 10^5^ Da, respectively. The results showed that the molecular weight of DGPIIa was slightly decreased compared with that of DGPIa after the removal of the protein. As reported, animal polysaccharides usually contain a large amount of protein, which is linked to the sugar residue mainly through N-glycopeptide bonds and/or O-glycopeptide bonds. The β-elimination reaction is usually used to detect the connection type between sugar and protein. The absorbance values at 240 nm of the polysaccharide containing N-glycopeptide bonds do not change after the reaction, while the absorbance values of the polysaccharide containing O-glycopeptide bonds increase significantly after the reaction (Pang et al., 2022). As shown in Figure 2D, after the β-elimination reaction, the A240 nm value of DGPIa increased markedly, indicating the presence of an O-glycopeptide bond. The monosaccharide compositions of DGPIa and DGPIIa were examined by a combination of PMP derivation and HPLC analysis (Figure 3A). According to the results, DGPIa and DGPIIa were composed of Man, GlcA, GalNAc, Glc, Gal, Fuc, with the molar ratios of were 1: 4.22 : 1.55: 0.18 : 8.05: 0.83 (DGPIa) and 0.74 : 1.78: 1: 0.28: 5.37 : 0.36 (DGPIIa), respectively. Wang et al. obtained a neutral polysaccharide from the Chinese frog *Rana chensinensis* (RCSPII) by hot water extraction and ion-exchange chromatography and found that RCSPII consisted of Glc (95.32%), Gal (3.01%), and Man (1.67%). In this work, we obtained an acid heteropolysaccharide (DGPIIa) from the skin of *Rana dybowskii* Guenther, which consists of 10.5% GalNAc and 18.68% GlcA. In addition, a small amount of Fuc (3.78%) was also detected. Polysaccharides, as biomacromolecules, have complex physicochemical properties and structures, which may be affected by the animal or plant source, spatial arrangement, genus, geographic region (Li et al., 2018), and extraction and purification methods (Zhu et al., 2016).

**TABLE 1:** Chemical composition (%) of DGP, DGPI and DGPII.

Polysaccharide	Total sugar (%)	Protein (%)	Uronic acid (%)	SO_4_^2−^ (%)
DGP	13.35 ± 0.85	19.93 ± 1.48	14.51 ± 0.72	3.50 ± 0.37
DGPI	24.61 ± 1.27	7.32 ± 1.39	16.87 ± 1.53	4.16 ± 0.83
DGPII	59.40 ± 1.51	4.36 ± 0.56	17.25 ± 1.89	4.63 ± 0.47

### 3.2 FT-IR analysis

The FT-IR spectra of DGPIa and DGPIIa are shown in Figure 3B. The results showed that they both showed the characteristic signal of polysaccharides. The absorption peak of the O^−^H group was observed at 3386 cm^-1^ and 3397 cm^-1^; the absorption peak at 2936 cm^-1^ is derived from the strong stretching vibration of C^−^H (Jiang et al., 2020), and the absorption peaks at approximately 1653 cm^-1^ and 1544 cm^-1^ indicate the presence of acetylamine (^−^NH_2_COCH_3_) (Mou et al., 2017); the characteristic absorption peak at 1408 cm^-1^ was attributed to ^−^COO^-^ (Shi et al., 2021); the absorption peaks at 1245 cm^-1^ and 1238 cm^-1^ were attributed to the stretching vibration of S^=^O (Yang et al., 2018); and the weak absorption peak at 1076 cm^-1^ in DGPIa or 1068 cm^-1^ in DGPIIa indicates that DGPIa and DGPIIa glycosidic bonds are of pyran type (Li et al., 2023). In summary, O^−^H, ^−^C^-^H bond, ^−^NH_2_COCH_3_ and ^−^COO^-^ are the characteristic groups of aminosaccharides, and it is presumed that DGPIa and DGPIIa may be glycosaminoglycans.

### 3.3 Effects of DGPIIa on learning and memory

**FIGURE 4:**
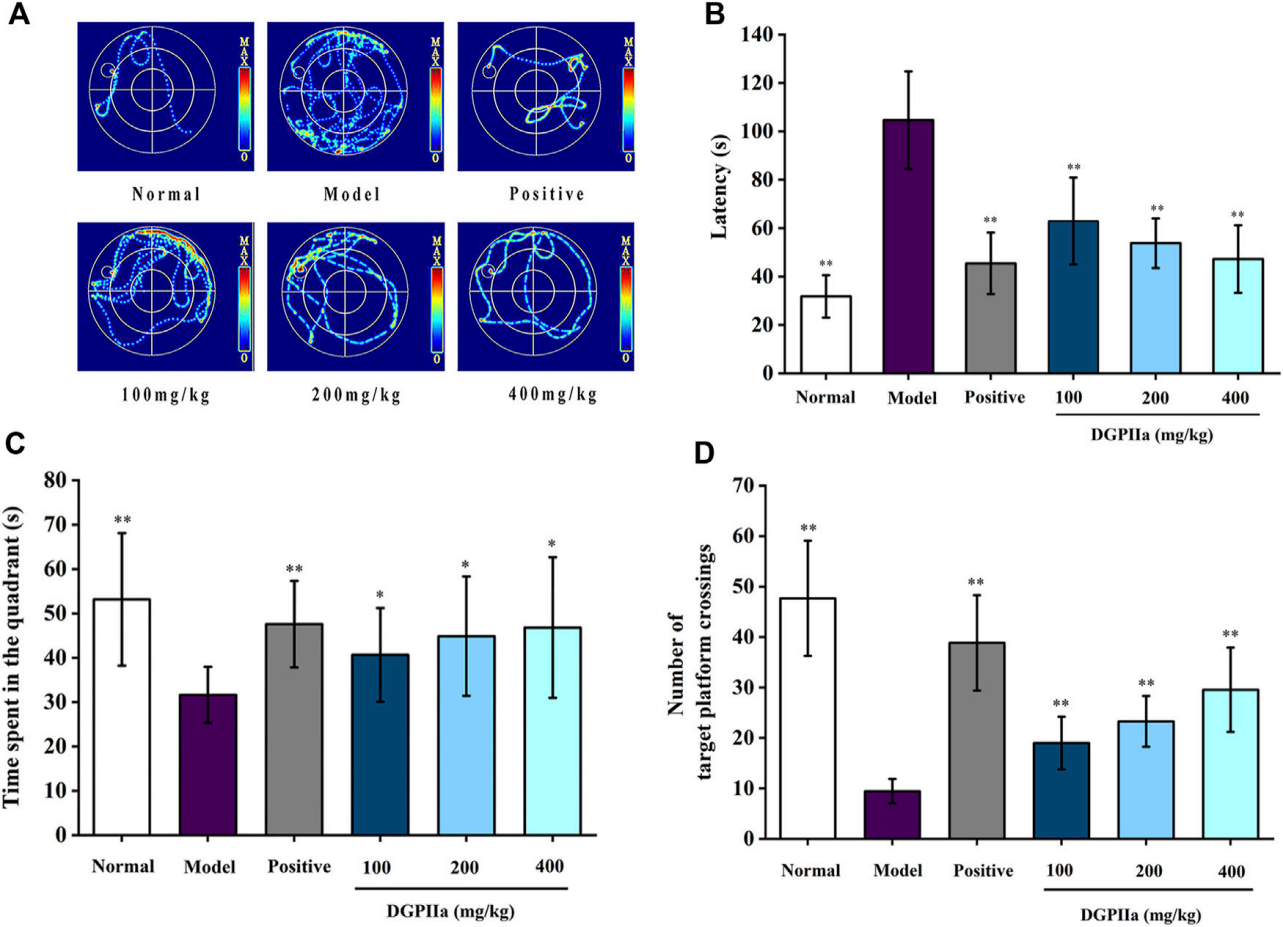
Effect of DGPIIa on learning and memory ability in morris water maze test. **(A)** the swimming track of experimental rats on day 5; **(B)** Escape latency to find the platform; **(C)** Time spent in the quadrant; **(D)** Number of target platform crossings. Data were expressed as mean ± SD. *p < 0.05, **p < 0.01, compared with aging model control group induced by D-gal.

D-gal accelerates aging in rodents, leading to a decrease in their learning and memory abilities. Therefore, D-gal-treated mice are commonly used to evaluate and screen anti-aging active natural polysaccharides. The Morris water maze is a classic method for evaluating the learning and memory abilities of animals. In this work, a D-gal-induced aging mouse model was used to investigate the anti-aging effects of DGPIIa, and we first used the Morris water maze test to assess the effect of DGPIIa on spatial learning and memory ability in aging mice. Compared with the normal control group, the mice in the model control group had a significantly longer escape latency (*p* < 0.01), indicating that D-Gal treatment impaired the spatial learning memory ability of mice (Figures 4A, B). In contrast, intervention with DGPIIa effectively and dose-dependently improved the learning ability of aging mice. The escape latency of mice in the 100, 200 and 400 mg/kg DGPIIa-administered groups was shortened by 39.82%, 48.59%, and 54.85%, respectively, and there was a significant difference between the DGPIIa-treated groups and the model control group (*p* < 0.01). In the spatial exploration experiment, two metrics were examined: the time the mice stayed in the target quadrant and the number of times the mice crossed the platform. As shown in Figures 4C, D, mice in the model control group had significantly reduced time spent in the target quadrant and number of platform crossings in the target quadrant compared with the normal control group (*p* < 0.01), indicating again that the memory retention ability of mice in the model control group became weaker. Compared with the model control group, the time spent in the target quadrant of DGPIIa-treated mice increased by 28.48% (100 mg/kg), 41.79% (200 mg/kg), and 47.89% (400 mg/kg) (*p* < 0.05). The number of mice that crossed the target platform dose-dependently increased, and the optimal effect was 2.13 times that of the model control group. These results indicated that DGPIIa enhanced the memory and learning abilities of aging mice.

### 3.4 Histopathology analysis of the brain

**FIGURE 5:**
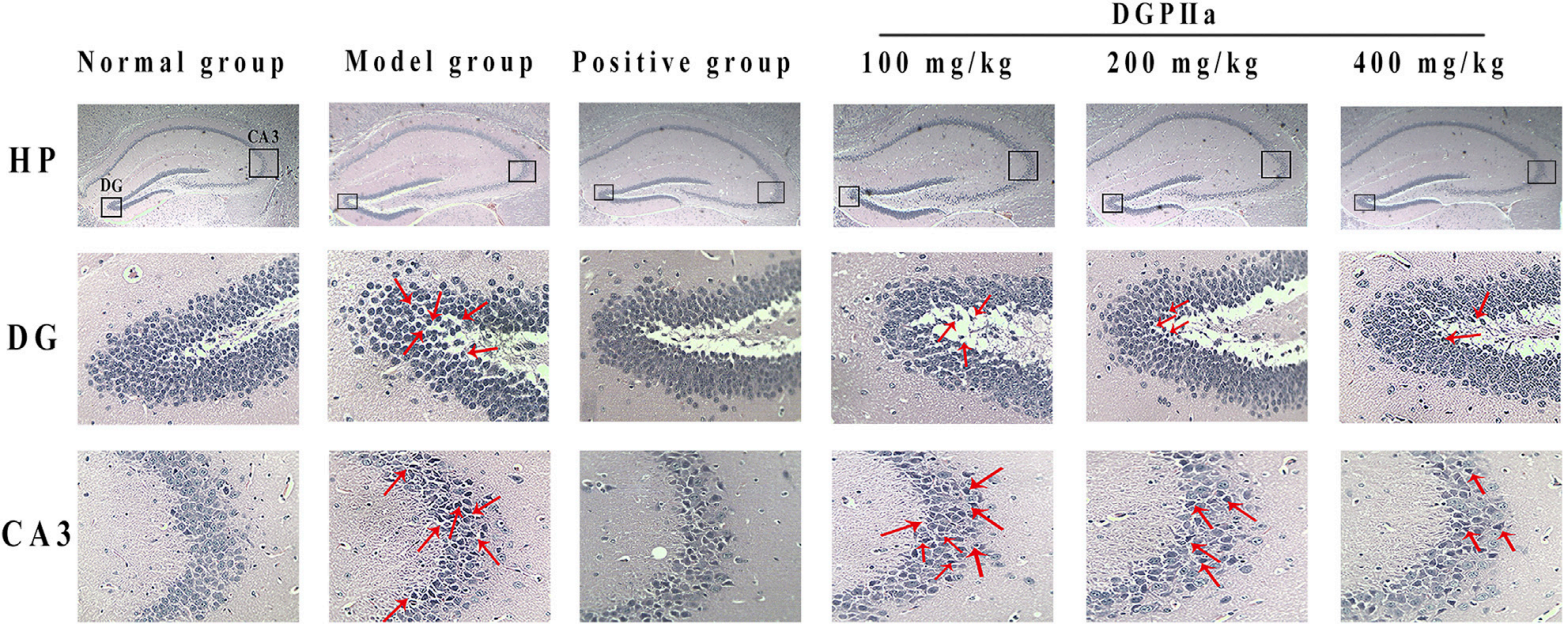
Effects of DGPIIa on morphological changes of brain tissues by H & E staining.

In this experiment, H & E staining was used for the preliminary observation of the pathological morphology of mouse brain tissue (Figure 5). The hippocampal cells in the normal control group were full, round and neatly arranged, with a large number of cells, clear nuclear membranes and lightly stained nuclei. However, the number of hippocampal cells in the model control group was significantly reduced, the arrangement was irregular and looser, some neurons showed obvious morphological changes, such as nuclear pyknosis, dark staining and even vacuolization, and more gaps appeared. Under D-Gal induction, the structural morphology of mouse hippocampal cells was damaged, and the brain was subjected to severe oxidative stress. In contrast, after treatment with DGPIIa (especially at doses of 200 and 400 mg/kg), there was a significant improvement in the histopathological changes in the brain of the mice, the morphology of neurons gradually returned to normal, the cells were arranged tightly and neatly with clear nuclei, and the improvement effect of the 400 mg/kg group was optimal.

### 3.5 Effect of DGPIIa on antioxidant enzyme activity in D-gal-induced aging mice

In the current study, D-Gal was widely used to establish an aging model in mice, which can cause the accumulation of ROS, indirectly stimulate the production of free radicals, and contribute to the production of oxidative stress (Wu et al., 2016). Excessive intake of D-Gal causes apoptosis and mitochondrial dysfunction, resulting in aging effects in multiple organs, and is often used to screen drugs with antioxidant and anti-aging effects. Studies have shown that D-gal-induced senescence may inhibit the PI3K/Akt pathway and inactivate Nrf2-mediated antioxidants in the liver, resulting in reduced CAT and SOD protein expression (Zhao et al., 2019). Antioxidant properties and anti-aging activity are closely related, and oxidative stress is the core mechanism that induces aging-related diseases, which is caused by a disruption in the balance between intracellular ROS production and removal (Zhang et al., 2011). Antioxidant enzymes, including SOD, GSH-Px and CAT, can counteract ROS generated *in vivo* during oxidative stress and act synergistically at different sites of the free radical metabolic pathway (Cai et al., 2018). SOD decomposes superoxide anion radicals into hydrogen peroxide, while GSH-Px and CAT catalyze the reduction of H_2_O_2_ to H_2_O and O_2_ and impede the production of hydroxyl radicals (Nataraj et al., 2022). MDA is a natural product of lipid peroxidation (Liu et al., 2020). The chain reaction between free radicals generated by lipid peroxidation and polyunsaturated fatty acids will decrease the fluidity of the cell membrane and impede cell metabolism, thus accelerating oxidative stress and the aging process of the body (Abuja and Albertini, 2001).

As shown in Table 2, the body mass of the mice in the model control group was significantly reduced, while the mice in the DGPIIa group that received different concentrations of DGPIIa showed significant improvement in body mass. In the concentration range of 100–400 mg/kg, compared with the model control group, the thymic index and the spleen index dose-dependently increased, and 400 mg/kg DGPIIa treatment showed the best effect, with an increase of 49.72% for the thymic index and 32.33% for the spleen index (*p* < 0.01; *p* < 0.05).

**TABLE 2:** Effects of DGPIIa administration on body weight (B.W.) and the immune organ indexes of mice.

Groups	Initial B.W. (g)	Finial B.W. (g)	Thymus index (%)	Spleen index (%)
Normal group	27.45 ± 0.79	47.51 ± 2.06	0.212 ± 0.032**	0.203 ± 0.025
Model group	26.24 ± 1.37	38.75 ± 2.51	0.153 ± 0.018	0.176 ± 0.033
Positive group	25.95 ± 0.66	39.94 ± 2.69	0.236 ± 0.017 **	0.231 ± 0.022**
DGPIIa (100 mg/kg)	25.88 ± 1.17	40.80 ± 2.92	0.188 ± 0.023**	0.174 ± 0.032
DGPIIa (200 mg/kg)	26.16 ± 1.18	40.49 ± 4.04	0.213 ± 0.024**	0.212 ± 0.036*
DGPIIa (400 mg/kg)	26.40 ± 1.17	41.39 ± 2.27	0.229 ± 0.018**	0.233 ± 0.037**

Data were expressed as mean ± S.D. (n = 10).

B.W.: body weight.

*p < 0.05, **p < 0.01, compared with aging model control group induced by D-gal.

**FIGURE 6:**
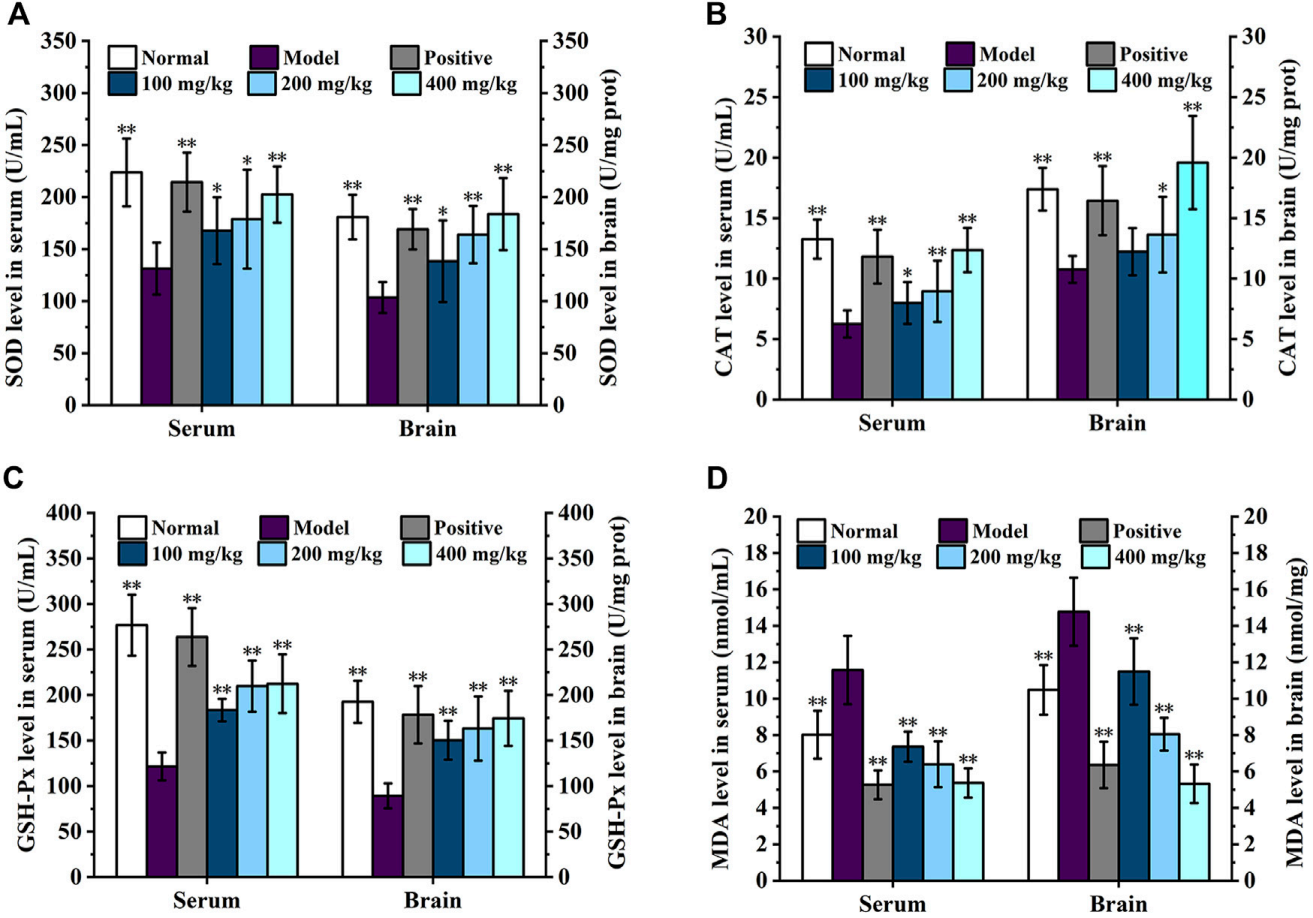
Effect of DGPIIa on antioxidant property of D-gal-induced aging mice. **(A)** SOD activity in serum and brain; **(B)** CAT activity in serum and brain; **(C)** GSH-Px activity in serum and brain; **(D)** MDA content in serum and brain. Data were expressed as mean ± SD. *p < 0.05, **p < 0.01, compared with aging model control group induced by D-gal.

The effects of DGPIIa on the activities of SOD, CAT, and GSH-Px and the level of MDA in mouse serum are shown in Figure 6. Compared with the normal control group, the SOD, GSH-Px and CAT activities in the serum of aging mice were significantly decreased (*p* < 0.01; *p* < 0.05), and the level of MDA was increased, indicating that oxidative damage in mice was caused by continuous injection of D-gal. In contrast, DGPIIa intervention significantly and dose-dependently increased SOD, GSH-Px and CAT activities (*p* < 0.01; *p* < 0.05) and decreased the level of MDA in the serum of aging mice. At the dose of 400 mg/kg DGPIIa, SOD, CAT and GSH-Px activities were increased by 54.17%, 97.63% and 74.68%, respectively, and the level of MDA was decreased by 53.53% compared with those of the model control group. In addition, the SOD activity tended to be similar to that of the positive control group.

The effects of DGPIIa on the activities of SOD, CAT, and GSH-Px and the level of MDA in the brain are shown in Figure 6. Consistent with previous results, in the concentration range of 100–400 mg/kg, the activities of SOD, GSH-Px and CAT were significantly increased compared with those in the model control group (*p* < 0.01; *p* < 0.05), while the MDA content was significantly lower than that in the model control group in a dose-dependent manner (*p* < 0.01), and the 400 mg/kg group showed the best effect. Compared with the model control group, the activities of SOD, CAT and GSH-Px in the 400 mg/kg group were increased by 77.46%, 82.01% and 95.30%, respectively, and the level of MDA was decreased by 64.01%. These results indicated that DGPIIa effectively alleviated oxidative damage and improved the antioxidant ability of aging mice.

Polysaccharides have great antioxidant potential, and a large number of studies have shown that a variety of plants have antioxidant effects (Wang et al., 2016). Glycosaminoglycans and chitin in animal polysaccharides have been shown to have antioxidant properties. Younes et al. prepared chitosan by N-deacetylation of chitin obtained from shrimp shells, which exhibited molecular weight-dependent antioxidant activity (Younes et al., 2014). Glycosaminoglycans are mainly categorized as chondroitin sulfate, hyaluronic acid dermatan sulfate, etc. Previously, chondroitin sulfate RPCS were extracted from *Raja porosa cartilage* by Zhou et al. also showed good free radical scavenging ability, probably due to the presence of sulfate groups in chondroitin sulfate (Zhou et al., 2020).

The structural features of polysaccharide are closely related to its bioactivities. Studies have shown that the monosaccharide composition is a key reason affecting the antioxidant activity of polysaccharides. Li et al. found that the uronic acid content, including GlcA and GalA, had important effect on the free radical scavenging activity of polysaccharides Li et al. (2015). The research of Jiang et al. shown that a galactan in *Mentha haplocalyx* Briq exhibites significant antioxidant and anti-aging activities (Jiang et al., 2020). Tang et al. obtained three exopolysaccharide fractions (r-EPS1, r-EPS2, and r-EPS3) from *Lactobacillus delbrueckii* ssp. *bulgaricus* SRFM-1 and found that their antioxidant activity was positively correlated with Gal content (Tang et al., 2017). Furthermore, animal polysaccharides typically contain amount of GalNAc, which is also an important factor affecting polysaccharide activity. Previously, Yang et al. reported that GalNAc-containing chondroitin sulfate extracted from the bones of *Oreochromis niloticus* had significant antioxidant activity (Yang J. et al., 2023). A glycosaminoglycan (DGPIIa) was also obtained in our study. The results were consistent with the reports of previous study, which conformed that DGPIIa containing mainly of GalNAc and Gal has strong antioxidant and anti-aging activity. However, it has been shown that the antioxidant activity of polysaccharides may also be affected by factors such as molecular weight, chemical composition, and structure (Xu et al., 2011), so this conformational relationship needs to be further investigated.

## 4 Conclusion

In summary, the acid polysaccharide DGPIIa was prepared from the skin of *Rana dybowskii* Guenther by water extraction, gel chromatography and deproteinization treatment. Structural analysis showed that DGPIIa was composed of Man, GlcA, GalNAc, Glc, Gal, and Fuc. The Morris water maze test showed that *Rana dybowskii* Guenther polysaccharides can improve learning and memory in D-Gal-induced aging mice; it could also repair pathological changes in the hippocampal region of the brain to some extent. In addition, DGPIIa significantly enhanced SOD, CAT, and GSH-Px activities and reduced MDA levels in serum and brain tissues in aging mice. Therefore, DGPIIa had an ameliorative effect on the aging-induced decline in learning and memory capacity. Our results provide useful data for the use of *Rana dybowskii* Guenther skin polysaccharides as a natural antioxidant and in anti-aging products.

## Data Availability

The original contributions presented in the study are included in the article/Supplementary material, further inquiries can be directed to the corresponding author.

## Abbreviations

Ara, arabinose; CAT, catalase; D-Gal, D-Galactose; FT-IR, Fourier transform infrared; Fuc, fucose; Gal, galactose; GalA, galacturonic acid; GalNAc, N-acetylgalactosamine; Glc, glucose; GlcA, glucuronic acid; GlcNAc, N-acetylglucosamine; GSH-Px, glutathione peroxidase; H&E, hematoxylin-eosin; HPGPC, high-performance gel-permeation chromatography; Man, mannose; MDA, malondialdehyde; Mw, molecular weight; PMP, 1-phenyl-3-methyl-5-pyrazolone; RDG, *Rana dybowskii* Guenther; RDGP, *Rana dybowskii* Guenther polysaccharides; Rha, rhamnose; ROS, reactive oxygen species; SOD, superoxide dismutase; UV, Ultraviolet; VC, vitamin C; Xyl, xylose.
